# P-78. Susceptibility of Omadacycline in Bone and Joint Infections: Pathogen Susceptibility and Regimen Decisions from an Ongoing Randomized Controlled Trial

**DOI:** 10.1093/ofid/ofaf695.307

**Published:** 2026-01-11

**Authors:** Amy Y Kang, Evelyn A Flores, Donna Phan Tran, Buchen (Olivia) Han, Isabel Payan, Guarina Garcia Delgado, Loren G Miller

**Affiliations:** Chapman University / Harbor-UCLA Medical Center, Irvine, CA; Division of Infectious Diseases, the Lundquist Institute at Harbor-UCLA Medical Center, Torrance, CA, Torrance, California; Division of Infectious Diseases, the Lundquist Institute at Harbor-UCLA Medical Center, Torrance, CA, Torrance, California; Lundquist Institute for Biomedical Innovation at Harbor-UCLA Medical Center, Torrance, California; The Lundquist Institute, COMPTON, California; Lundquist Institute at Harbor-UCLA Medical Center, Claremont, California; Lundquist Institute at Harbor-UCLA Medical Center, Claremont, California

## Abstract

**Background:**

Treatment of Bone and joint infections (BJIs) with oral antibiotics may have benefits compared to IV therapy. Yet oral treatment options may be limited by lack of options due to antibiotic-resistant pathogens and tolerability concerns. Omadacycline has *in vitro* activity against common BJI pathogens, including MRSA and most Enterobacterales. However, real-world susceptibility data and implications for oral therapy are limited.

**Table 1.**
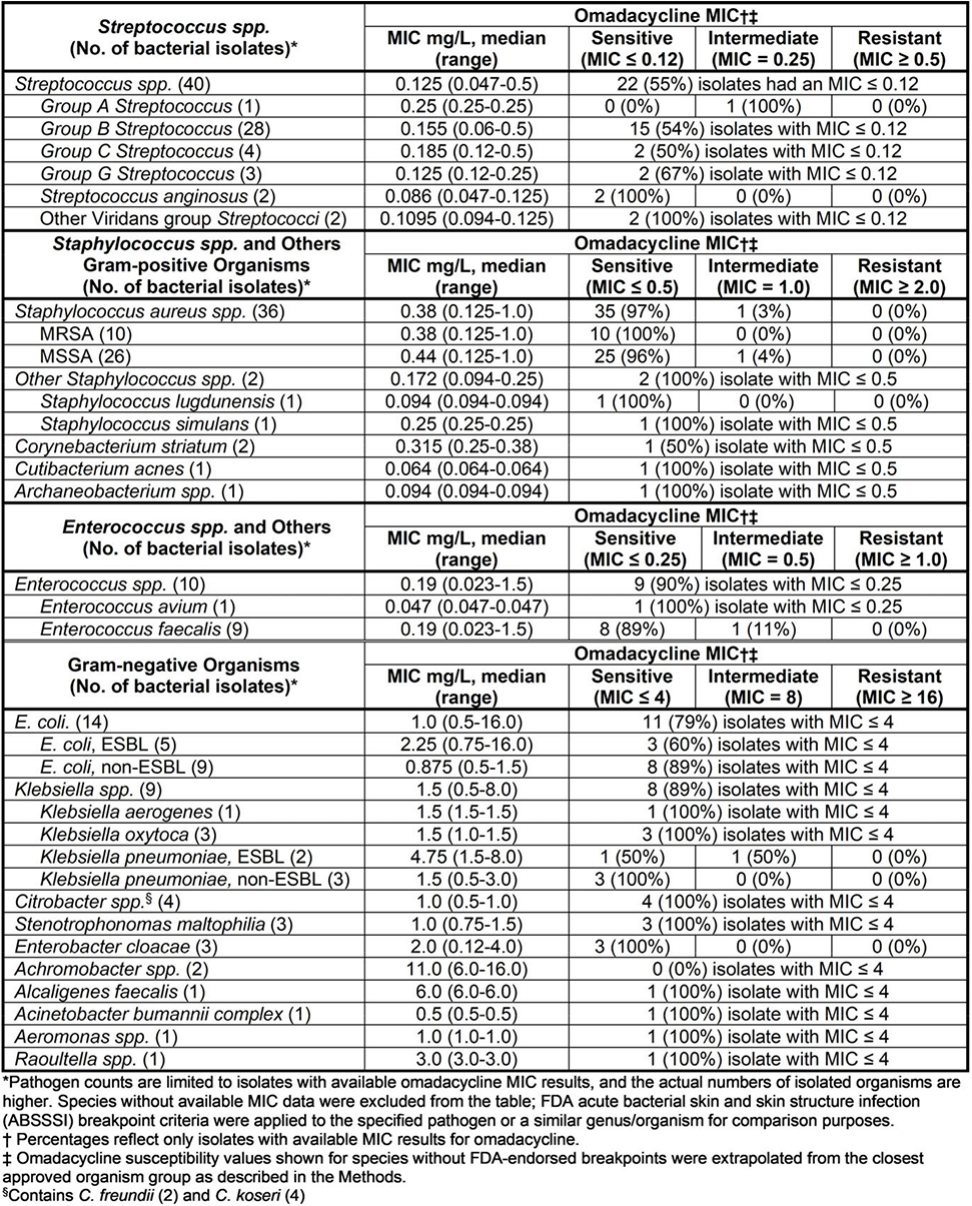
Omadacycline In-vitro Susceptibility in Bone and Joint Infections among All Screened Patients

**Table 2.**
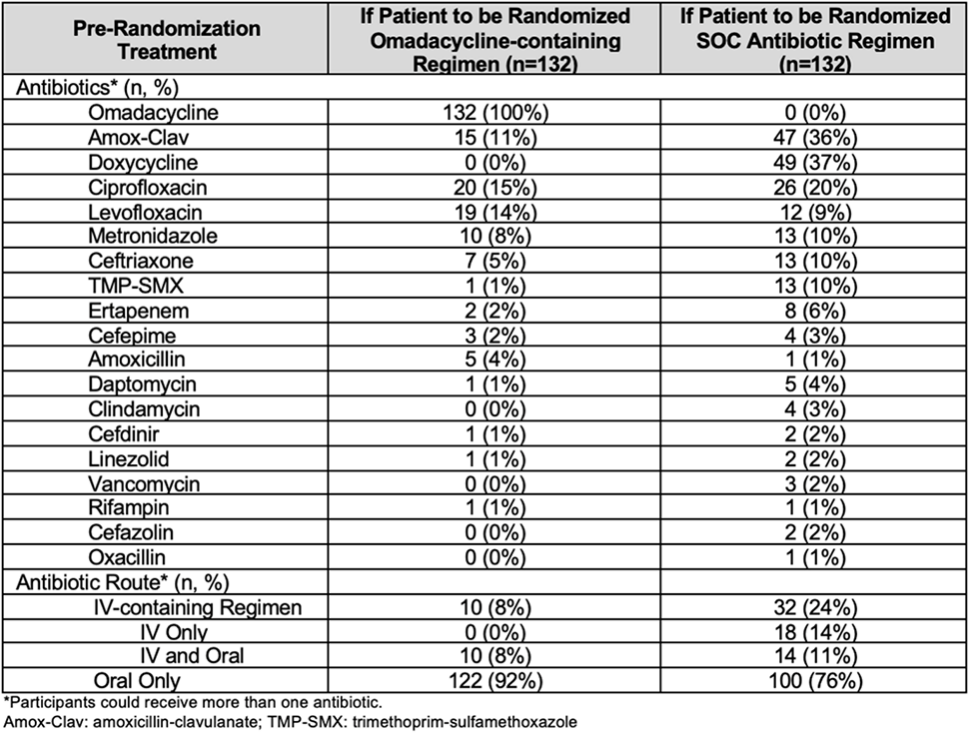
Pre-Randomization Treatment Choices

**Methods:**

We conducted a descriptive analysis of data from adult patients enrolled to date in an open-label, randomized controlled trial of comparing omadacycline-containing antibiotic regimen vs. standard of care (SOC) antibiotics for BJIs. Omadaycline susceptibility was assessed on available clinical isolates using MIC Test Strips (Liofilchem®). Susceptibility to omadacycline was interpreted using FDA breakpoints where available or extrapolated for organisms without criteria. We also described omadacycline-containing vs. SOC regimens (provider-chosen) among randomized patients to assess the proportion of oral vs. IV therapies.

**Results:**

To date, we screened 162 patients and randomized 132 (81%). Most randomized patients had diabetic foot infections (113/132 (86%)). Among screened patients, bacterial isolates were recovered from 144 (89%) participants. The most frequently identified isolates were *Streptococcus* spp. (37%), *S. aureus* (22%), 24% of which were MRSA, followed by and Enterobaterales (19%). Susceptibility data are summarized in Table 1. Among randomized patients and prior to randomization, 122/132 (92%) of omadacycline contating regimens would be oral-only therapy, compared to 100/132 (76%) in the SOC arm (p< 0.001).

**Conclusion:**

In our randomized trial of BJI treatment, omadacycline demonstrated in vitro activity against most BJI pathogens, including *Streptococcus*, MRSA and Enterobacterales. A higher proportion of omadacycline-containing regimens were eligible for oral-only therapy compared to SOC. In light of these findings, omadacycline may warrant consideration as an oral option in select BJI cases

**Disclosures:**

Amy Y. Kang, Pharm.D., BCIDP, ACCP: Grant/Research Support|Paratek Pharmaceuticals: Grant/Research Support|SIDP: Grant/Research Support Loren G. Miller, MD MPH, Armata: Grant/Research Support|GSK: Grant/Research Support|Merck: Grant/Research Support|Paratek: Grant/Research Support

